# Does NUCB2/Nesfatin-1 Influence Eating Behaviors in Obese Patients with Binge Eating Disorder? Toward a Neurobiological Pathway

**DOI:** 10.3390/nu15020348

**Published:** 2023-01-10

**Authors:** Mariarita Caroleo, Elvira Anna Carbone, Biagio Arcidiacono, Marta Greco, Amedeo Primerano, Maria Mirabelli, Gilda Fazia, Marianna Rania, Marta Letizia Hribal, Luca Gallelli, Daniela Patrizia Foti, Pasquale De Fazio, Cristina Segura-Garcia, Antonio Brunetti

**Affiliations:** 1 Department of Health Sciences, University Magna Graecia of Catanzaro, 88100 Catanzaro, Italy; 2 Department of Medical and Surgical Sciences, University Magna Graecia of Catanzaro, 88100 Catanzaro, Italy; 3 School of Medicine, Cardiff University, Cardiff CF14 4XN, UK; 4 University Hospital Mater Domini of Catanzaro, 88100 Catanzaro, Italy; 5 Department of Experimental and Clinical Medicine, University Magna Graecia of Catanzaro, 88100 Catanzaro, Italy

**Keywords:** nesfatin-1, NUCB2, obese, eating disorders, polymorphisms, binge eating, eating behaviors

## Abstract

Nesfatin-1 is a new anorexigenic neuropeptide involved in the regulation of hunger/satiety, eating, and affective disorders. We aimed to investigate nesfatin-1 secretion in vitro, in murine adipose cells, and in human adipose fat samples, as well as to assess the link between circulating nesfatin-1 levels, *NUCB2* and Fat Mass and Obesity Gene (*FTO*) polymorphisms, BMI, Eating Disorders (EDs), and pathological behaviors. Nesfatin-1 secretion was evaluated both in normoxic fully differentiated 3T3-L1 mouse adipocytes and after incubation under hypoxic conditions for 24 h. Omental Visceral Adipose tissue (VAT) specimens of 11 obese subjects, and nesfatin-1 serum levels’ evaluation, eating behaviors, NUCB2 rs757081, and FTO rs9939609 polymorphisms of 71 outpatients seeking treatment for EDs with different Body Mass Index (BMI) were studied. Significantly higher levels of nesfatin-1 were detected in hypoxic 3T3-L1 cultured adipocytes compared to normoxic ones. Nesfatin-1 was highly detectable in the VAT of obese compared to normal-weight subjects. Nesfatin-1 serum levels did not vary according to BMI, sex, and EDs diagnosis, but correlations with grazing; emotional, sweet, and binge eating; hyperphagia; social eating; childhood obesity were evident. Obese subjects with CG genotype NUCB2 rs757081 and AT genotype FTO rs9939609 polymorphisms had higher nesfatin-1 levels. It could represent a new biomarker of EDs comorbidity among obese patients.

## 1. Introduction

Obesity is one of the most common nutritional disorders, and its medical, psychological, social, and economic effects have a significant burden on healthcare resources [1], leading to high rates of morbidity and mortality worldwide [2]. The development of obesity has multifactorial causes, so the interaction among multiple genes, as well as environmental factors and behaviors, has an impact on body weight and eating habits [3]. The comorbidity rate between obesity and Eating Disorders (EDs) is high [4,5]. In particular, the lifetime prevalence of obesity in EDs is reported to be 28% on average, ranging from 5% in Anorexia Nervosa (AN) to 87% in Binge Eating Disorder (BED) [6]. In this light, different phenotypes of obese patients in relation to eating dysfunctional behaviors were recently described [7]. Many biomarkers were analyzed to explain the variability in eating behaviors and the biological mechanisms [8] that could affect the brain response to food intake [9]. The hypothalamus, the main regulating center for energy balance, is abundant with peptides that regulate satiety [10]. Many of these peptides have also been described in peripheral sites (e.g., adipose tissue) to be important in regulating body weight homeostasis and providing the rise and progression of the metabolic abnormalities observed in obese patients [11]. A newly discovered neuropeptide hormone, nesfatin-1, has been recently associated with the development of obesity [12,13,14]. Studies on lung cell lines provided, for the first time, information on nesfatin-1 [15], disclosing the expression of leptin receptors succeeding the activation by the PPAR-γ ligand troglitazone. This molecule was identified as an anorexigenic peptide derived from its precursor non-esterified fatty acid (NEFA)/nucleobindin-2 (*NUCB2*) protein after post-translational cleavage by specific convertases, PC2 and PC3/1 [15,16]. 

The cleavage of *NUCB2* by PCs in other potential sites produces the nesfatin-2 (residues 85–163) and nesfatin-3 (residues 166–396) forms, but only nesfatin-1 or the full-length *NUCB2* has the potential role to induce satiety [17]. Furthermore, when *NUCB2* is proteolytically transformed into nesfatin-1, it can stimulate anorexia [17]. Polymorphisms in the *NUCB2* gene have been associated with susceptibility to obesity [18]. 

Nesfatin-1 is abundantly secreted both in central and peripheral regions. It has been detected in several regions of the hypothalamus and demonstrated to play key roles in the control of food intake, while it seems to be modulated by starvation and refeeding [19,20]. As nesfatin-1 has been demonstrated to cross the blood–brain barrier bidirectionally [21], it has been hypothesized that peripheral nesfatin-1 might enter into the brain and modulate appetite and feeding response [22]. Recently, the gastrointestinal tract was also found to be an abundant source of peripheral nesfatin-1, suggesting its ability to improve mucosa regeneration, to restore the balance between pro-oxidants and antioxidants mechanisms, to interplay with the gut microbiota, and to modulate the structure of the intestinal barrier [23]. Another potential source of peripheral nesfatin-1 is adipose tissue, which influences the integration of metabolic activity and energy balance by communicating, via adipokines, with peripheral organs and the brain [24]. Nesfatin-1 is a good candidate to be part of the brain–adipose axis [25]. Circulating nesfatin-1, which is supposed to be derived from peripheral tissues, might play a role in the transmission of anorexigenic signals from the periphery to the brain [26]. Many studies aimed at understanding the role of nesfatin-1 in the development of obesity or diabetes point out inconsistent results. This may be due to the short life-time of circulating nesfatin-1 (* 10–20 min) [27]. To date, the principal origin of circulating nesfatin-1, its receptor, the pharmaco-modulation of signaling, and the regulation of nesfatin-1 production in the periphery are still unknown [19]. The possible relationship between nesfatin-1 and Body Mass Index (BMI) is yet uncertain and studies have shown conflicting results, with positive [28,29,30,31], negative [32,33], or no [34] correlation. In addition, a growing body of recent evidence has suggested nesfatin-1 as a regulator of emotional stress responses [35], affective [36,37], and EDs [38], but results are still controversial. Despite an increasing number of neurochemical studies, there is not enough data on the effect of central nesfatinergic signaling on the course of affective and EDs [39]. Furthermore, there are no fully appropriate yet available experimental animal models. In consideration of its pleiotropic functions, as well as with regard to its dual hypothalamic and peripheral sites of synthesis and localization, our hypothesis was that nesfatin-1 could play a role in food intake behaviors among obese patients in relation to the co-occurrence of ED and/or affective diseases. BMI, eating behaviors, anxiety, or depressive symptoms could, in fact, hypothetically, influence nesfatin-1 levels, both in Visceral Adipose Tissue (VAT) and in the bloodstream. 

To our knowledge, this is the first study that compared nesfatin-1 levels according to eating behaviors and psychopathological features among patients at different BMI ranges, with or without an eating disorder. We aimed to investigate differences in NUCB2/nesfatin-1 levels in human VAT and in serum of obese patients compared to normal and underweight controls. Second, we tested the nesfatin-1 secretion profile in in vitro models of fully differentiated 3T3-L1 adipocytes. In order to estimate the link between obesity development and nesfatin-1 levels, polymorphisms of NUCB2/nesfatin-1 and the Fat mass and obesity gene (*FTO*) were also assessed. Finally, circulating nesfatin-1 plasma levels were evaluated in human subjects across a range of BMI and were compared to eating behaviors and affective symptoms.

## 2. Materials and Methods

### 2.1. 3T3-L1 Cell Culture and Differentiation

3T3-L1 murine preadipocytes were cultured and differentiated as previously described [40]. Cells were cultured in serum-free DMEM containing 0.5% bovine serum albumin (BSA) and incubated in the hypoxic glove chamber with 1% O_2_/5% CO_2_, at 37 °C for 48 h [41]. As a surrogate system for the study of adipose cell dysfunction in obesity, once differentiated, mature 3T3-L1 adipocytes were incubated at 37 °C under hypoxic conditions (2% O_2_) for 24 h [42]. Control cells were maintained under the same conditions, but in normal atmosphere (21% O_2_). At the end of incubation, the chamber was opened in the anaerobic glove box (flushed with N_2_) to avoid reoxygenation. Cell viability was tested by 3-(4,5-dimethythiazol-2-yl)-2,5-diphenyl tetrazolium bromide assay, to evaluate the absence of cytotoxicity after exposure to hypoxia, as described previously [43]. 

### 2.2. Adipose Tissue Biopsies from Human Subjects

Omental VAT specimens were obtained from 11 consecutive, unrelated, obese subjects, during elective bariatric surgery, and 6 normal-weight controls from patients undergoing open abdominal surgery. Each specimen was immediately snap-frozen in liquid nitrogen and stored at −80 °C until protein extraction. All the operated subjects were nonsmokers and had not taken any medications for at least 6 months before the study. Criteria of exclusion were secondary obesity, genetic syndromes of insulin resistance, psychiatric disorders, Type 1 or Type 2 diabetes, previous gestational diabetes mellitus, hepatic or renal impairment, presence of malignancies or rheumatologic disease, and treatment with medications affecting glucose tolerance. 

### 2.3. Western Blot Analyses

Total protein extracts were prepared from cells and tissues as previously described [44]. Briefly, cells were washed twice with PBS and then detached with a rubber policeman in 1 mL of cold PBS. The cell suspension was transferred in a 1.5 mL tube and pelleted by centrifugation at 9000 rpm for 3 min at 4 °C. Proteins were extracted with cold Lysis buffer (137 mM NaCl, 20 mM Tris-HCl, pH 7.6, 1 mM MgCl_2_, 1 mM CaCl_2_, 1,5% NP-40, 2 mM EDTA) supplemented with the protease inhibitor cocktail, PMSF (2 mM), and phosphatase inhibitor. The final concentration in the extracts was determined using the Bradford colorimetric assay (Biorad). Proteins (20 µg) were separated by sodium dodecyl sulfate polyacrylamide gel electrophoresis (SDS-PAGE), transferred to a PVDF membrane (Immobilon-PSQ 0.2 µm Millipore ISEQ00010), and blotted for 2 h. Blotted membranes were treated with blocking solution (1XTBS, 0.1% Tween-20) with 5% *w*/*v* nonfat dry milk, and then incubated overnight at 4 °C with anti NUCB2/nesfatin-1 (1:1000 dilution; code no. NBP2-35072; Novus Biologicals), or anti beta Actin antibodies. Anti-rabbit (1:2000; P0448; Dako Denmark A/S) or anti mouse IgG horseradish conjugates (1:2000; P0447; Dako Denmark A/S) were used as secondary detection antibodies. Immune complexes were visualized by enhanced chemiluminescence (ECL, Amersham) and autoradiographic films, and the density of specific bands was measured using the ImageJ software program. 

### 2.4. Patient Enrollment and Clinical Characterization

A total of 71 consecutive outpatients seeking treatment for EDs were recruited at the University “Magna Graecia” of Catanzaro, Unit of Psychiatry, from September 2017 to July 2019. Participants were considered eligible if they were: 18–65 years old; drug-naïve; diagnosed with AN restrictive type or BED according to DSM-5, suffering from obesity without an ED in comorbidity or normal-weight healthy controls without any psychiatric diagnosis according to DSM-5; able to answer a self-reporting questionnaire; able to understand the process in which they were involved. Obesity was defined as BMI > 30 kg/m^2^ based on the World Health Organization (WHO) [45]. For each participant, data regarding physiological features, pharmacological treatments, and current and past history of mental and physical disorders were collected. Those people aged under 18 or above 65 years; patients diagnosed with AN purging type or BN according to DSM-5; normal-weight individuals with any comorbid psychiatric condition as coded by DSM-5; patients with Type 1 or Type 2 diabetes, neurological conditions or other medical disorders that could exert some type of action on the cognitive functioning of the person; hormonal and pharmacological treatment potentially able to induce either cognitive impairment or metabolic changes; pregnancy or childbirth over the previous 12 months; smokers were left out from the recruitment. In addition to clinical examinations, patients were subjected to blood withdrawal for the determination of circulating nesfatin-1 level and genotyping. The study protocol and procedures complied with ethical principles set out in the Helsinki Declaration. The study obtained the approval by local Ethics Committee (Comitato Etico Regione Calabria, sezione Area Centro: 565/D.G. 1 August 2017). Each participant signed a written informed consent before any procedure took place. 

### 2.5. Anthropometric Measurements

Were measured using.

A portable stadiometer (Seca 220, Seca GmbH & Co., Hamburg, Germany) and a balance scale (Seca 761) were used to measure participants’ height and weight and calculate BMI. Subjects were asked to wear light indoor clothing and no shoes. Standing height was measured to the nearest 0.1 cm and body weight to the nearest 0.1 kg at 8.00 a.m. BMI was classified according to the WHO guidelines [45]. 

### 2.6. Psychopathological Features

Trained psychiatrists interviewed participants by means of the Structured Clinical Interview for the DSM-5 (SCID-5-CV) [46] and the Eating Disorder Examination (EDE 17.0 D) [47] for the assessment of possible psychiatric and eating disorders, respectively. A thorough assessment of pathological eating behaviors (i.e., binge eating, emotional eating, grazing, sweet eating, craving for carbohydrates, night eating, post-dinner eating, hyperphagia, social eating, reducing portions, skipping meals, and long fasting) during the previous 6 months was also carried out. Pathological behaviors were deemed present in the case of both criteria: affirmative answer to all related items and clinically significant impairment or distress, as previously described [48]. Then, patients answered the following questionnaires:

- The 21-item self-report Beck Depression Inventory (BDI-II) [49] to evaluate depressive symptoms. Scores of 0–9, 10–16, 17–29, and ≥30 indicate minimum, mild, moderate, and severe depression, respectively. Cronbach’s alpha in the present research was 0.917. BDI-II score was used as a covariate in the statistical analysis.
- The Binge Eating Scale (BES) [50] is useful to evaluate binge-eating severity. Total BES scores <17, 17–27, and >27, respectively, indicate unlikely, possible, and probable risk of BED.
- The State-Trait Anxiety Disorder (STAI) [51]. This 40-items tool measures state (STAI-St) and trait (STAI-Tr) anxiety. Cronbach’s alpha for the data in this study was 0.845.

### 2.7. Determination of Patients’ Plasma Levels of Nesfatin-1

Venous blood collection was performed at around 08.00 a.m. after overnight fasting. Following venous blood withdrawal, blood was collected in pre-cooled laboratory EDTA tubes containing aprotinin (BD Vacutainer, K3E 15%, Aprotinin 250KIU, BD Plymouth. PL67BP; UK) to prevent protease activity. Tubes were then centrifuged at 4 °C for 10 min at 3000 g (rotor radius: max = 12.7 cm, min = 10.7 cm), and plasma aliquots were stored at −80 °C until use. Plasma nesfatin-1 was measured using a commercial enzyme-linked immunosorbent assay, and the Mybiosource human NES1 (Nesfatin-1) ELISA kit, according to the manufacturer’s instructions. The assay displayed a detection sensitivity of 9.38 pg/mL, a detection range of 15.63–1000 pg/mL, and a coefficient of variation < 10%. The antibody used in this ELISA was raised against nesfatin-1 and recognized both nesfatin-1 and the full-length protein precursor NUCB2 containing the epitope. 

### 2.8. Patient Genotyping

DNA was extracted from whole blood using commercial DNA isolation kits (Promega, Madison, WI and Roche, Mannheim, Germany). rs757081NUCB2/nesfatin-1 and rs9939609 FTO genotype calls were determined with the TaqMan allelic discrimination assay (Assay ID# C_2261417_10 and C_30090620_10 for NUCB2/nesfatin-1 and FTO, respectively, Applied Biosystems, Foster City, CA, USA). Template DNA was amplified, and fluorescence was detected on a BioRad CFX-96 Thermal Cycler (Bio-Rad Laboratories, Inc., Hercules, CA, USA). Genotyping quality was tested by including a blinded duplicate in each 96-well assay. The average agreement rate of duplicate samples was >99%.

### 2.9. Statistics

The data are reported as means, standard deviations (SD), frequencies, and percentages (%). Generalized linear model (GLM) analysis was conducted to compare the main effects of ED diagnosis and BMI categories and the interaction between EDs, BMI, and eating behaviors on plasma nesfatin-1 levels controlling for BES, BDI-II, and STAI. EDs diagnosis included four levels (healthy control, AN, BED, and obesity), while BMI consisted of five progressive levels (underweight, normal weight, and obesity classes I, II, and III). Forward stepwise linear regression analysis served to establish variables associated with nesfatin-1 considering BMI, age, sex, childhood obesity, BDI-II, and BES scores as independent variables. Two-tail Spearman correlations were run between eating behaviors and nesfatin-1 circulating levels. Statistical analysis was performed with the Statistical Package for Social Sciences version 21 (SPSS, Chicago, IL, USA).

## 3. Results

### 3.1. Nesfatin-1 Protein Secretion in Hypoxic 3T3-L1 Cells

**Figure 1:**
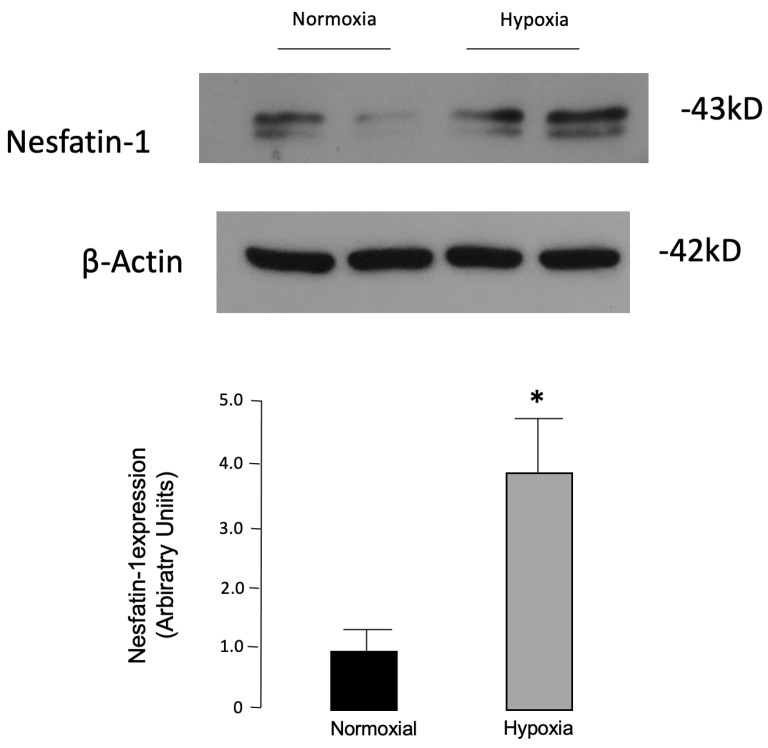
Effects of hypoxia on nesfatin-1 protein expression in murine 3T3-L1 adipocytes. Fully differentiated 3T3-L1 adipocytes were incubated under hypoxic conditions (2% O_2_) for 24 h maintained in normoxia (21% O_2_). Western blot analysis was performed with 3L3-L1 protein extracts using an anti-nesfatin antibody. Beta-actin antibody served as the protein loading control. Representative WBs of three separate experiments and mean densitometric analyses of WBs performed in 3T3-L1 cells under different oxygen tension are shown. * *p* < 0.003 vs. normoxia (Student’s *t*-test).

3T3-L1 murine differentiated adipose cells have been shown to secrete nesfatin-1 protein [28]. To establish whether nesfatin-1 production varies following hypoxia, a condition known to trigger adipose cell dysfunction in obesity, 3T3-L1 adipocytes were exposed for 24 h to hypoxic conditions (2% O_2_) [52] and compared to cells maintained in normoxia. Nesfatin-1 protein was detected at significantly higher levels in hypoxic 3T3-L1 cultured adipocytes as compared to normoxic control cells (Figure 1), suggesting that in obesity-related conditions, a similar finding could take place in human adipose cells. 

### 3.2. Nesfatin-1 Protein Secretion in Human Adipose Tissue

**Figure 2:**
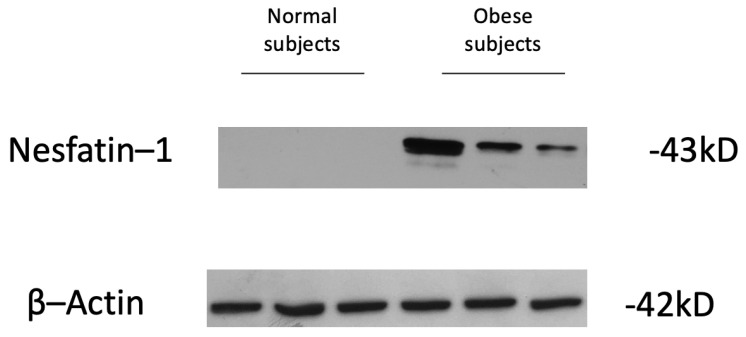
Nesfatin-1 protein expression in adipose tissue explants from obese and normal-weight subjects. Protein extracts from adipose tissues were loaded. Corresponding protein levels for nesfatin-1 were determined by Western blot analysis using anti-nesfatin-1 antibody as described in the Materials and Methods. β-actin was employed as a control of protein loading. A representative blot of three obese and three normal-weight patients is shown.

To investigate whether human adipose tissue indeed produces nesfatin-1, and to clarify whether this protein is more abundant in obese vs. non-obese subjects, protein extracts from omental VAT of obese and normal-weight patients were analyzed by Western blot analysis. Nesfatin-1 was searched in human VAT from controls (*n* = 6; mean BMI = 23.6 ± 1.5 kg/m^2^) and obese patients (*n* = 11; mean BMI = 45.8 ± 6.1 kg/m^2^). Our data showed that nesfatin-1 was undetectable in the VAT of normal-weight individuals, while a significant amount of this protein was observed in all (*n* = 11) obese patients (Figure 2), reinforcing the concept that nesfatin-1 may be differentially produced in adipose tissue in relation to patients’ BMI.

### 3.3. Demographic, Anthropometric, and Psychopathological Characteristics of Patients

A total of 71 subjects, aged 36.4 ± 12.7 years, were included in the current cross-sectional study. Table 1 summarizes the main descriptive and psychopathological tests. According to the WHO guidelines [45], 14 participants were underweight (BMI < 18.5 kg/m^2^), 16 normal weight (BMI 18.5–24.9 kg/m^2^), while 14 had class I (BMI 30–34.9 kg/m^2^), 6 class II (BMI 35–39.9 kg/m^2^), and 21 class III obesity (BMI > 40 kg/m^2^). For statistical purposes, patients with obesity were split into two groups: Obesity 1, corresponding to class I (BMI 30–35 kg/m^2^; *n* = 14); Obesity 2, corresponding to class II / III (BMI > 35 kg/m^2^; *n* = 27). No differences in sex distribution were observed upon BMI categories (χ^2^ = 4.445; *df* = 3; *p* = 0.217); instead, patients with AN and BED were overrepresented among females (χ^2^ = 13.058; *df* = 3; *p* = 0.005). 

### 3.4. Circulating Nesfatin-1 Levels in Human Plasma

**Figure 3:**
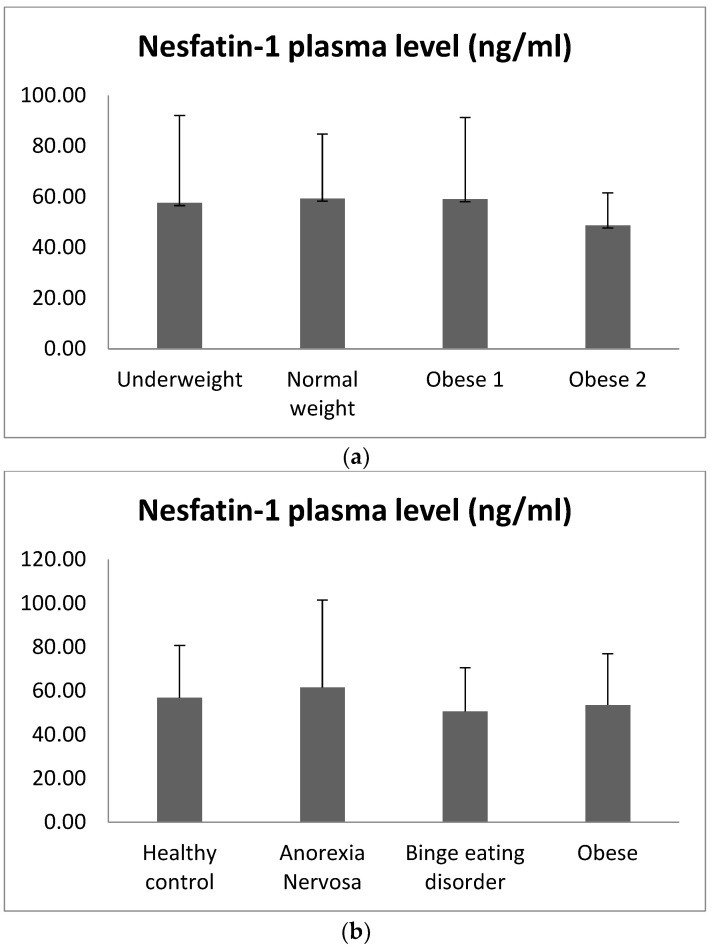
(**a**) Nesfatin-1 plasma levels according to BMI categories; (**b**) Nesfatin-1 serum levels according to EDs diagnosis.

Average levels of circulating nesfatin-1 are summarized in Table 2 and Figure 3a,b. Based on GLM, nesfatin-1 plasma levels did not vary in relation to the BMI category (F = 2.122; *p* = 0.130), EDs diagnosis (F = 0.244; *p* = 0.784), or sex (F = 0.917; *p* = 0.343).

Although no significant correlations emerged between nesfatin-1 and EDs diagnosis, inverse correlations with binge eating, grazing, emotional and sweet eating, and positive correlations with hyperphagia and social eating were evident (Table 3). 

Finally, a series of linear regression analyses was run to ascertain variables associated with nesfatin-1 considering BMI, age, sex, childhood obesity, BDI-II score, and BES score as independent variables (Table 4). Higher plasma levels of nesfatin-1 were correlated to childhood obesity and lower scores of the BES scale. 

### 3.5. Association between NUCB2 rs757081 and FTOrs9939609 Genetic Polymorphisms and Circulating Nesfatin-1

To search for further interrelationships between nesfatin-1 and the obese status, we analyzed circulating nesfatin-1 levels in obese patients genotyped for the most prevalent genetic polymorphisms for the *NUCB2/nesfatin-1* gene, and for *FTO* (FTO alpha-ketoglutarate-dependent dioxygenase), a known gene involved in the regulation of fat mass, adipogenesis, and body weight [53]. NUCB2 rs757081 and FTO rs9939609 were analyzed in a small subset of obese participants. Table 5 displays the distribution of alleles and genotypes in relation to nesfatin-1 rs757081 polymorphism and plasma levels of nesfatin-1. The distribution of alleles and genotypes in the groups was consistent with the Hardy–Weinberg equilibrium and comparable to other European populations, confirming a normal distribution. Although no significant differences were evident, obese subjects with the CG genotype had higher mean nesfatin-1 levels (F (2.23)1.258; *p* = 0.303). 

**Figure 4:**
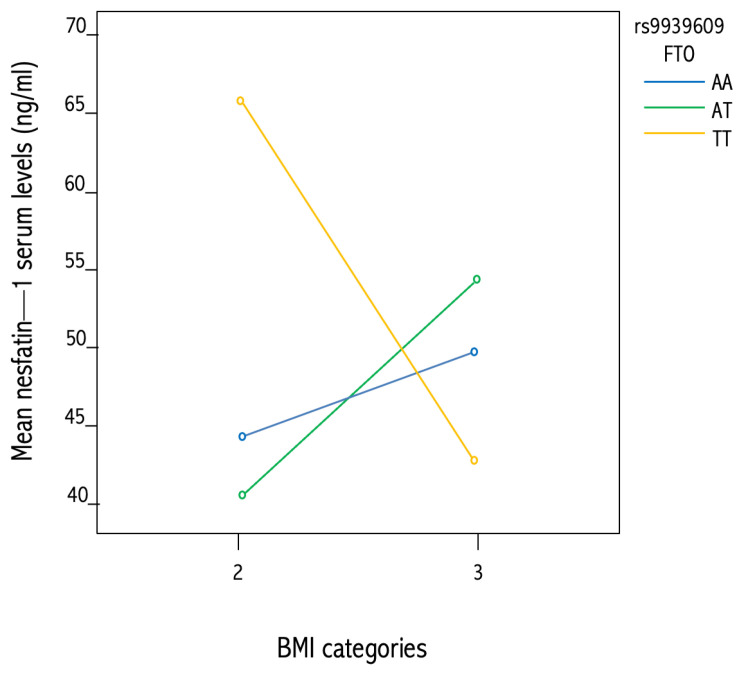
Interaction of FTO × BMI on nesfatin-1 plasma levels.

The genetic analysis for FTO rs9939609 polymorphisms showed that the AT genotype was the most frequent. GLM analysis demonstrated that a significant effect of the interaction between FTO × BMI emerged (F = 4.935; *p* = 0.018) on nesfatin-1 plasma levels (Table 6; Figure 4). 

## 4. Discussion

Nesfatin-1 has been identified as an anorexigenic peptide with multiple localizations, including adipose tissue. Whether this factor may play a role in obesity and eating behaviors is, however, poorly understood. We chose to address this issue by using a translational approach, and first analyzed 3T3-L1 cells, a murine adipose cell line commonly used to investigate the biology of adipose cells. It was previously demonstrated that nesfatin-1 is also secreted by cultured adipocytes, and its secretion levels vary according to the maturation state of adipocytes, although there exist mixed results. In these cells, nesfatin-1 has been previously shown to be synthesized and released into the culture medium in increasing concentrations during the differentiation from preadipocytes into adipocytes [28], while Tagaya et al. [54] reported the reduction in endogenous nesfatin-1 levels after the induction of 3T3-L1 cells differentiation. Consistent with the decrease in endogenous nesfatin-1 protein levels during 3T3-L1 adipogenesis, the stable knockdown of NUCB2 resulted in increased adipogenesis, while stable overexpression of NUCB2 decreased neutral lipid accumulation and reduced adipogenic gene expression [55]. Thus, despite the conflicting results, these findings hint that nesfatin-1 could contribute to the adipogenesis regulation. In consideration of this assessment, we determined nesfatin-1 protein levels in fully differentiated 3T3-L1 cultured adipocytes exposed to 24 h of hypoxia as a surrogate model of the molecular changes occurring in obesity [56]. In our study, significantly higher levels of nesfatin-1 were found in hypoxic vs. normoxic 3T3-L1 adipocytes. In previous reports, in the same in vitro model, it was shown that other adipokines, including visfatin, were induced by hypoxia [52], and similar findings were later confirmed in human adipose tissue [57]. Oh-I et al. [15], trying to identify new appetite-regulating molecules, proved that the nesfatin-1 precursor, NUCB2, was secreted by 3T3-L1 adipocytes and that its levels increased after treatment with troglitazone in rat hypothalamic and 3T3-L1 adipocyte cells.

Thus, we next examined nesfatin-1 in human VAT in relation to BMI. Previous findings confirmed the expression of nesfatin-1 in VAT [28,58,59], and even to a greater extent, in subcutaneous adipose tissue [28]. However, we opted to search the nesfatin-1 protein in VAT because, as a major endocrine organ, it produces a variety of bioactive molecules, whose abnormal expression is strongly associated with systemic, low-grade inflammation, and insulin resistance, two conditions linked to obesity and to obesity-related disorders [60,61]. In adipose tissue, nesfatin-1 has been shown to increase with obesity and to be modulated by feeding and starvation [59], while circulating and adipose tissue levels of nesfatin-1 were reported to be higher in diet-induced obese mice [28]. Hypoxia is a characteristic feature of the adipose tissue in obesity, playing a determinant role in changes in the adipokine profile associated with obesity progression [62,63]. Adipose tissue hypoxia is considered an important trigger of adipose cell dysfunction in both animal models and humans with obesity [44]. Although no previous study could confirm these results, we hypothesized that hypoxia could play a role in the overproduction of nesfatin-1 in VAT. Our data indeed demonstrated, for the first time, higher concentrations of nesfatin-1 in omental VAT of obese patients with respect to normal-weight controls, confirming a positive correlation between nesfatin-1 and BMI. Previous studies have demonstrated the presence of nesfatin-1 only in adipose tissue of normal-weight subjects [28]. Thus, nesfatin-1 could be a good candidate to play a role in the brain–adipose axis. Indeed, it was tested to be secreted in the hypothalamus and in both white and brown adipose tissue, and its adipose, hypothalamic, and circulating levels are altered by states of feeding and starvation, being decreased by starvation and increased after re-feeding or a high-fat diet [55]. 

As a further step forward in the understanding of potential bonds between nesfatin-1 and BMI, we also evaluated plasma levels of nesfatin-1 and rs757081 polymorphism. In this regard, many studies have, in fact, tried to explain the link between the *NUCB2/nesfatin-1* gene and the development of obesity [14]. Recently, Zegers et al. [14] posited that mutations and polymorphisms in the nesfatin-1 encoding gene *NUCB2* might cause obesity in humans, and identified genetic variants in obese individuals, suggesting that nesfatin-1 might indeed be involved in the regulation of energy homeostasis and food intake [14]. They analyzed a large sample of people with obesity and normal-weight controls, reporting an association between obesity and three Single-Nucleotide Polymorphisms (SNPs) (i.e., rs1330, rs214101, and rs757081). These SNPs were associated, only among males, with BMI, weight, and fat-free mass, leading to the concept that SNPs in the *NUCB2* gene could play an important role in the prediction of, and protection against, the development of obesity in male subjects. Chen et al. [64] independently demonstrated that the association of the *NUCB2* variant c.1012C > G (Q338E or rs757081) is linked to childhood adiposity, while the less frequent GG genotype (Q338E) could be considered as protective against excessive adiposity gain, providing further evidence for the role of *NUCB2* in the determination of human adiposity. To note, even if our data did not reach significance, their trend is in line with this study. We also examined the possible correlation between nesfatin-1 serum levels and the *FTO* gene variants. SNPs of the *FTO* gene are intensely associated with obesity [65]. Among these SNPs, the rs9939609 polymorphism exhibits a strong effect on BMI, body fat, and body composition [66,67]. The rs9939609 polymorphism A-allele was associated with higher caloric intake and eating a high-calorie diet [68]. The *FTO* gene plays an important role in fat metabolism, and the *FTO* risk allele carriers display lower fat cell lipolysis compared with the others [69]. The mechanism at the basis of the association between *FTO* and obesity development remains unclear. Some studies reported that the *FTO* gene may exert its effects by altering body composition and gaining adiposity [69,70]. Our results have shown that the AT genotype was the most frequent in our sample, and a significant effect of the interaction between the *FTO* gene and BMI emerged on nesfatin-1 serum levels. 

Despite this evidence, contradictory results have been reported regarding the association between BMI and circulating nesfatin-1 levels [28,29,30,31,32,33,34]. Initial lines of evidence showed a negative correlation between nesfatin-1 levels and BMI [32,71]. However, none of these studies included obese subjects. Conversely, a significant association between fat percentage and circulating nesfatin-1 in obese and morbid obese subjects was found [28,72]. In our study, we did not find significant differences in plasma nesfatin-1 concentration in relation to BMI. Although this finding seems to be counterintuitive and needs to be explained, discrepancies between protein secretion in tissues and in circles should be evaluated within the complexity of the systemic responses in pathophysiology, and the limits of the analytical process. In addition, similar dynamics have been reported previously for other analytes with additional localizations, in addition to adipose fat [73]. In this regard, as a “satiety molecule” secreted from adipose tissue, nesfatin-1 was described to cross the blood–brain barrier and to be secreted into the cerebrospinal fluid (CSF) [30]. We speculated that the higher plasma nesfatin-1 levels observed in our obese subjects could be a consequence of reduced nesfatin-1 uptake into the CSF, possibly due to saturation of transporters [29]. While confirming this hypothesis, Tan et al. [30] reported significantly lower CSF/plasma ratios of nesfatin-1 that negatively correlated with BMI, body weight, and fat mass in obese adult subjects. 

We also described that higher circulating nesfatin-1 could be linked to childhood obesity. Recent studies agreed with these results [34]. In particular, Anwar et al. [31] also demonstrated that plasma nesfatin-1 levels were significantly higher in obese children and adolescents than in control subjects. Controversial results may point toward other factors that influence circulating nesfatin-1 levels. For example, the postprandial level of nesfatin-1 and its relationship with various clinical and metabolic parameters were also discussed [74]. It was demonstrated that fasting nesfatin-1 levels, after an oral glucose load, were not higher in obese than in healthy children, thus suggesting that oral glucose load may not be a proper test to induce the nesfatin-1 response and, conversely, that nesfatin-1 may not have an impact as a short-term regulator of food intake among obese children [74]. Long-term changes in body weight could probably affect nesfatin-1 levels with reported higher levels in overweight and obese men and women [28] and lower levels in female anorexic patients [75], resulting in a positive correlation with BMI. Although further confirmation is still needed in humans, in rats, the regulation of circulating nesfatin-1 appears to be dependent on food intake with lower levels after fasting and the restoration after re-feeding [28]. Concerning fasting plasma nesfatin-1 and its relation to dietary habits and daily intake, a study correlated nesfatin-1 positively with the percentage of calories derived from daily carbohydrates and saturated fat intake, and negatively with calories derived from daily protein intake [31]. Thus, the evaluation of eating behaviors seemed to be an important issue, and nesfatin-1 could be a useful biomarker of eating habits among obese patients. Our aim was, therefore, to identify recurring eating behaviors among obese patients that could be linked to plasma nesfatin-1 levels. In terms of trends, an inverse correlation emerged with binge eating, grazing, emotional and sweet eating, and craving for carbohydrates, frequently described as dysfunctional eating behaviors in obese patients with BED [7], and a positive correlation with hyperphagia and social eating, frequently described among obese patients without EDs [7]. We determined that higher levels of nesfatin-1 were associated with lower scores of the BES. A previous study confirms our results [13]. Although there are no studies to date that could explain this inverse relationship between binge eating and nesfatin-1 levels, we could consider important findings in other species to interpret our results. NUCB2/nesfatin-1 mRNA levels significantly decreased after a high-glucose and high-fat diet [76], such as during binge eating. Macronutrients could exert a modulatory effect on preproghrelin and NUCB2/nesfatin-1 mRNA and protein secretion in the intestine and hepatopancreas of goldfish in a time- and concentration-dependent manner [76]. This could be due to a distinct effect on nesfatin-1 transcription, translation, and post-translational processing. Thus, glucose induces changes not only at the mRNA level but also in the process that regulates the cleavage of nesfatin-1 from its precursor. Diet composition is considered an important factor for the regulation of metabolic hormones. In mammals, numerous studies have detailed a modulation of appetite-regulating hormones secretion, including ghrelin and nesfatin-1, by the macronutrient composition of diets [77], independently to BMI.

Obese patients with higher scores of the BES, probably affected by BED, have shown lower levels of nesfatin-1, so nesfatin-1 could be related to the comorbidity of EDs in obese patients. In this regard, lower plasma nesfatin-1, by altering the satiety signaling, could be the trigger and the maintaining determinant of binge behaviors. Moreover, coherently, low circulating nesfatin-1 has been defined as a facilitating factor for the development of obesity [78]. It is not yet clear if this variability in nesfatin-1 levels could account for the development and self-perpetuating of eating-related maladaptive behaviors. Chen et al. [79] shed new and intriguing light on the hypothesis about the role of nesfatin-1 in the genesis of EDs, suggesting its direct action on dopaminergic reward circuitries. It was recently described that the nesfatin neurons in the lateral amygdala send their inhibitory efferent branches to the VTA neurons, which result in the anorexigenic effect [39].

Few studies have been conducted to evaluate nesfatin-1 plasma levels in patients with EDs [38,39,75]. We did not find a significant correlation between plasma nesfatin-1 levels and AN. Previous studies demonstrated decreased circulating levels of nesfatin-1 in patients diagnosed with restricting-type AN [38,75], but mixed findings have been reported in obese subjects, with increased [80] or decreased [81] levels. Moreover, a hormone with anorexigenic and anxiogenic effects might play a role in the etiology or maintenance of AN, which is often accompanied by depression and anxiety [82]. Hoffman et al. [37] investigated the relationship among nesfatin-1 plasma levels, anxiety, depressiveness, and perceived stress in obese men and women and their alterations during inpatient treatment. Nesfatin-1 levels in plasma positively correlated with perceived anxiety and may also change in the course of an eating disorder [39]. However, we have not found significant differences in BDI-II and STAI scores among groups. Previous results have shown higher nesfatin-1 levels in women with high anxiety levels [12]. A positive correlation of nesfatin-1 with depression was also reported [36]. Our data have shown that eating behaviors could play a role in determining changes in plasma nesfatin levels rather than affective symptoms or BMI. Although previous results have shown that nesfatin-1 might be regulated in a sex-specific manner [12,83,84], our data do not demonstrate significant differences in circulating nesfatin-1 levels according to gender. Similarly, Anwar et al. did not show a significant difference in nesfatin-1 levels in relation to sex, in the obese group [31]. However, men with anxiety disorder displayed decreased plasma nesfatin-1 levels compared to healthy controls [84]. These findings could suggest a different secretion of nesfatin-1 in males and females affected by affective symptoms that warrants further research to investigate this possible sex-specific effect.

### Limits

Several limitations should be noted, prior to proceeding with the conclusions. First, the sample size limited the power in this study, and due to its exploratory nature, no corrections for multiple testing were made, thus increasing the likelihood of significant results. Second, the presence of nesfatin-1 in the adipose tissue of patients with or without BED was not verified, and it would be interesting to understand if nesfatin-1 levels also vary in relation to the reduction in binge behaviors. Overall, future investigations on nesfatin-1 on EDs need to ensure that such confounders are evaluated and described, and if possible, accounted for in statistical analyses. Limitations of cross-sectional studies obviate conclusions regarding causality.

Future studies with a longitudinal design could be useful to elucidate the variability in nesfatin-1 levels according to other variables, including the determination of nesfatin-1 in CSF in patients with varying degrees of BMIs.

## 5. Conclusions

The newly identified neuropeptide nesfatin-1 is characterized by a wide range of regulating activity in the brain and in other peripheral sites. Increasing evidence supports it as a new, potentially significant factor involved in the pathogenesis of mental disorders. To summarize, based on our results, in adipose tissue, nesfatin-1 abundance varied in relation to BMI. Higher levels were found in the VAT of obese patients with respect to normal-weight controls. Circulating nesfatin-1 levels could be influenced by other important variables. Our results showed that eating behaviors could play a role in determining plasma nesfatin-1 concentration rather than affective symptoms or BMI. An inverse correlation emerged with dysfunctional eating behaviors, such as binge eating, grazing, emotional and sweet eating, and craving for carbohydrates, frequently described in obese patients with BED, and a positive correlation with hyperphagia and social eating, described among obese patients without EDs.

In this regard, nesfatin-1 could be identified as a potential biomarker of comorbidity of EDs among obese patients.

## Figures and Tables

**Table 1:** Sample description and psychopathological tests.

		Mean	SD
**Age**		36.4	12.7
		***Fr***	***%***
**Sex**	F	45	63.4
	M	26	36.6
**BMI**	Underweight	14	19.7
	Normal weight	16	22.5
	Obese 1	14	19.7
	Obese 2	27	38.0
**Diagnosis**	Healthy control	20	28.2
	Anorexia nervosa	10	14.1
	Binge eating disorder	17	23.9
	Obese	24	33.8
**BDI-II**		16.1	12.3
**STAI**	State	43.3	13.3
	Trait	45.4	13.0
**BES**		12.4	11.7

Abbreviations: BMI, body mass index; BDI-II, Beck Depression Inventory-II; STAI, State-Trait Anxiety Inventory; BES, Binge Eating Scale.

**Table 2:** Nesfatin-1 plasma levels (ng/mL).

		Mean	SD
**BMI**	Underweight	57.54	34.582
	Normal weight	59.28	25.448
	Obese 1	59.05	32.268
	Obese 2	48.70	12.894
**Diagnosis**	Healthy control	56.93	23.847
	Anorexia nervosa	61.54	39.913
	Binge eating disorder	50.60	20.013
	Obese	53.58	23.355
**Sex**	F	53.31	24.843
	M	57.88	26.678

BMI, body mass index. Obesity 1 corresponds to Obesity class I; Obesity 2 corresponds to Obesity classes II and III.

**Table 3:** Correlations between nesfatin-1 and eating behaviors.

		*Nesfatin-1 (ng/mL)*
**Hyperphagia**	r	0.119
	*p*	0.478
**Binge**	r	−0.033
	*p*	0.844
**Grazing**	r	−0.123
	*p*	0.462
**Emotional eating**	r	0.031
	*p*	0.852
**Post-dinner eating**	r	−0.218
	*p*	0.188
**Night-eating**	r	−0.015
	*p*	0.928
**Sweet eating**	r	−0.050
	*p*	0.767
**Social eating**	r	0.180
	*p*	0.279
**Craving for carbohydrates**	r	−0.073
	*p*	0.663

r: Spearman correlation between plasma nesfatin-1 levels and altered eating behaviors; *p*: statistical value, *p* < 0.05 is considered significant.

**Table 4:** Linear regression analysis.

Variable		Non-Standardized Coefficients		Standardized Coefficients	t	*p*
		B	SD Error	β		
1	(Constant)	1.765	0.140		12.603	0.000
	Child obesity	0.599	0.283	0.307	2.115	0.040
2	(Constant)	2.096	0.166		12.590	0.000
	Child obesity	0.852	0.271	0.437	3.144	0.003
	BES	−0.030	0.010	−0.431	−3.103	0.003

BES, Binge Eating Scale; SD, standard deviation. Dependent variable: nesfatin-1 serum levels. *p*: *p* value is significant if <0.05.

**Table 5:** Mean plasma nesfatin-1 levels (ng/mL) based on NUCB2 polymorphism.

rs757081	Mean	N	SD
*CC*	49.01	12	13.468
*CG*	52.90	11	12.494
*GG*	40.10	3	5.157
Total	49.63	26	12.655

**Table 6:** Mean plasma nesfatin-1 levels (ng/mL) based on FTO polymorphism.

rs9939609	Mean	N	SD
*AA*	47.52	5	4.619
*AT*	50.51	14	12.569
*TT*	49.37	7	17.470
Total	54.99	26	25.432

## Data Availability

The data presented in this study are available on request from the corresponding author.
